# Anti-NMDA Receptor Encephalitis: A Narrative Review

**DOI:** 10.3390/brainsci15050518

**Published:** 2025-05-18

**Authors:** Vlad Pădureanu, Dalia Dop, Rodica Pădureanu, Denisa Floriana Vasilica Pîrșcoveanu, Gabriela Olaru, Ioana Streata, Ana Maria Bugă

**Affiliations:** 1 Department of Internal Medicine, University of Medicine and Pharmacy Craiova, 200349 Craiova, Romania; vlad.padureanu@umfcv.ro; 2 Department of Pediatrics, University of Medicine and Pharmacy Craiova, 200349 Craiova, Romania; 3 Department of Neurology, University of Medicine and Pharmacy Craiova, 200349 Craiova, Romania; 4 Doctoral School, University of Medicine and Pharmacy Craiova, 200349 Craiova, Romania; olarugabriela03@yahoo.com; 5 Department of Molecular Biology, University of Medicine and Pharmacy Craiova, 200349 Craiova, Romania; 6 Department of Biochemistry, University of Medicine and Pharmacy Craiova, 200349 Craiova, Romania

**Keywords:** encephalitis, NMDA receptor, molecular pathogenesis, diagnosis

## Abstract

Antibodies against the NR1 or NR2 subunits of the NMDA receptor are linked to anti-N-methyl-D-aspartate (NMDA) receptor encephalitis, a type of encephalitis that mainly affects women. Clinicians who treat patients of all ages should be aware of this type of encephalitis since it may be a treatable differential for symptoms and indicators observed in neurology and psychiatric clinics. Auditory and visual hallucinations, delusions, altered behavior (often accompanied by agitation), reduced consciousness, motor disruption (from dyskinesia to catatonia), seizures, and autonomic dysfunction are typical clinical characteristics. In recent years, the incidence of autoimmune encephalitis diagnoses has markedly risen among adults, children, and adolescents. This fact is unequivocally connected to the dynamic evolution of novel diagnostic techniques and the advancement of medical knowledge. A specific variant of this illness is anti-NMDA receptor encephalitis. Psychiatrists frequently serve as the initial specialists to treat patients with this diagnosis, owing to the manifestation of psychiatric symptoms associated with the condition. The differential diagnosis is quite challenging and predominantly relies on the patient’s history and the manifestation of characteristic clinical signs. Given its high prevalence, anti-NMDA receptor encephalitis should be included in the differential diagnosis in routine psychiatric treatment. We provide an overview of the research on the condition, covering its prognosis, management, epidemiology, differential diagnosis, and clinical presentation.

## 1. Introduction

Anti-N-methyl-D-aspartate (NMDA) receptor encephalitis first systematically described in 2007 by Dalmau and colleagues, who determined that 12 patients had significant neuropsychiatric symptoms. The individuals shared comparable neurological symptoms, including autonomic instability, dyskinesia, seizures, memory loss, psychosis, and diminished consciousness [1]. Ovarian teratomas were present in seven of these patients. Originally thought to be a paraneoplastic encephalitis, the scientists came up with the moniker “anti-NMDAR encephalitis” [1]. Most anti-NMDAR encephalitis cases in adolescents and young adults, particularly females in the first four decades of life, with or without a related tumor, were found in a follow-up study conducted by the same investigators [2]. It is a rare but increasingly recognized autoimmune disorder of the central nervous system (CNS) [1]. They were all found to have antibodies to the NMDA receptor in their serum or cerebrospinal fluid (CSF) [3]. Early symptoms, such as fever and headache, may resemble the flu. Patients may go into a coma in severe cases [4]. About 15% of patients may relapse during the acute phase, which is severe and has a 5% fatality rate [5,6]. In 2018, 633 patients with anti-NMDAR encephalitis had their clinical symptoms compiled in a systematic review [7]. It is characterized by the production of pathogenic antibodies against the NMDA receptor (NMDAR), predominantly targeting the GluN1 subunit, leading to a constellation of psychiatric and neurological symptoms due to glutamatergic neurons cell signaling impairment. Over the past decade, significant advances have been made in understanding the clinical manifestations, pathophysiology, diagnostic strategies, and management of this condition. To improve the prognosis and comorbidities of anti-NMDAR encephalitis and lower the chance of relapse, the 2021 International Consensus suggests immunosuppression, plasma exchange, hormonal therapy, early intervention, and symptomatic treatment [8]. However, there is still disagreement on and a lack of understanding of the therapeutic mechanisms of anti-NMDAR encephalitis.

Since many patients with anti-NMDAR encephalitis exhibit prominent mental symptoms, including as anxiety, agitation, sadness, manic episodes, unusual behavior, delusional or paranoid thinking, and visual or auditory hallucinations, the condition might be mistakenly diagnosed as other psychiatric disorders [9,10]. There have been reports of certain patients with the presence of anti-NMDAR antibodies in the cerebrospinal fluid (CSF) at the time of their initial schizophrenia diagnosis [11,12]. Additionally, it was reported that immunotherapy plus anti-inflammatory medication worked well for a patient with anti-NMDAR antibodies who had been diagnosed with treatment-resistant schizophrenia [13]. Psychiatric or cognitive symptoms, without seizures, involuntary movements, hypoventilation, or related tumors, may be the primary manifestations of atypical NMDAR encephalitis which can be easily misdiagnosed [14]. Nowadays, the differential diagnostics of anti-NMDAR encephalitis remain challenging.

The purpose of this narrative review is to provide a concise overview of anti-NMDA receptor encephalitis and to summarize the current state of knowledge.

## 2. Anti-NMDAR Encephalitis Epidemiological Data

Anti-NMDA receptor encephalitis is an autoimmune disorder of the central nervous system (CNS) that has been reported in individuals ranging from infancy to older adulthood; however, it predominantly affects young adults and children, with a female predominance [3]. Although frequently associated with ovarian teratomas (particularly in women of childbearing age), the disease can also occur in males or younger children, and in the absence of an identifiable tumor [1,15]. The true incidence of anti-NMDA receptor encephalitis is likely underestimated due to the variability in recognition and diagnostic methods; however, it is now recognized as one of the most common autoimmune encephalitis. After acute demyelinating encephalitis, epidemiological research indicates that anti-NMDA receptor encephalitis might be the most frequent cause of autoimmune encephalitis [16]. Although prevalence rates have not yet been estimated, over 500 cases have been documented [2,15,17]. Steiner et al. looked for several NMDA receptor antibodies in 121 people with schizophrenia. Two of them had the immunoglobulin G (IgG) NR1a antibodies of anti-NMDA receptor encephalitis, and about 10% (*n* = 15) tested positive for anti-NMDA receptor antibodies [11]. Zandi et al. [18] found that 3 out of 46 patients with first-onset schizophrenia had blood antibodies against the NMDA receptor. Therefore, it is still unclear how common anti-NMDA receptor encephalitis is in the general population and in those with psychosis. Anti-NMDAR encephalitis appears to occur more frequently than any other known paraneoplastic encephalitis, although the precise prevalence is unknown [2]. The median age of the disease beginning was 22.6 years (ranging between 8 months to 84 years), according to a recent review that included demographic studies in 633 patients. Of these patients, 492 were female (77.8%) and 141 were male (22.2%) [7]. It was found that 133 cases (21.0%) had a viral prodrome at the start of symptoms, including fever (56 cases) and headache (75 cases) [7]. Of all the patients, 178 cases (28.1%) had ovarian teratoma, and 28 cases (4.4%) had additional malignant neoplasms [7]. Compared to those without a teratoma, those with a teratoma had a considerably higher likelihood of presenting with mental symptoms, psychosis, and autonomic symptoms [7]. Whether tumors other than teratomas are pathogenic of the disease or unrelated concurrent illnesses is yet unclear [2].

## 3. Anti-NMDAR Encephalitis Molecular Pathogenesis

The hallmark of this disease is the generation of autoantibodies in the brain that target the extracellular domain of the GluN1 subunit of the NMDA receptor [1,19]. NMDARs are crucial for synaptic transmission, synaptic plasticity, and higher-order cognitive functions, including memory and learning. The dysfunction or internalization of these receptors due to antibody binding leads to a characteristic spectrum of neuropsychiatric manifestations.

While the immunological triggers are not completely understood, they can be related to genetic or environmental factors, but many cases do not have a clear associated trigger. A well-documented association exists between anti-NMDA receptor encephalitis and ovarian teratomas, which often contain neural tissue that may prompt an autoimmune response via molecular mimicry [4]. Nevertheless, a substantial proportion of cases are considered non-paraneoplastic, with no underlying tumor identified. In both paraneoplastic and non-paraneoplastic cases, the production of anti-NMDAR autoantibodies reflects a dysregulated humoral immune response that targets neuronal antigens and disrupts normal synaptic function.

It seems that the presence of a tumor, primarily an ovarian teratoma that contains tissue from the nervous system and expresses NMDAR, is what causes anti-NMDAR encephalitis [20]. For a non-para-neoplastic cause of anti-NMDAR encephalitis, the bacteria and viruses are the most common reported triggers. Among the viral encephalitis, the herpes simplex virus (HSV) is the most reported trigger. The patients’ serum and cerebrospinal fluid (CSF) contain autoantibodies, which typically exhibit intrathecal production and high antibody concentrations [9]. NMDARs are heteromers of the glutamate-binding NR2 subunits and the glycine-binding NR1 subunits [21]. The N-terminal extracellular region of NR1 is recognized by the antibodies of all patients, indicating an antibody-mediated etiology [9,22]. The primary cause of anti-NMDAR encephalitis is NMDAR internalization without complement activation [2,9,15]. The internalization of NMDAR occurs through antibody binding, capping, and the cross-linking of the receptors, and the loss of the NMDAR from the cell surface correlates with the antibody titer [20]. A decreased synaptic NMDAR content leads to reduced synaptic plasticity, as demonstrated by the report that the treatment of rodent neurons with patient CSF blocks the molecular signatures of long-term potentiation (LTP) in the hippocampus [23]. According to a prior study, mice given patient antibodies by chronic cerebroventricular infusion develop gradual memory impairments, anhedonia, and behaviors resembling depression [24]. Restoring NMDAR levels was linked to a progressive clinical improvement following the cessation of the antibody infusion [25].

## 4. Is Anti-NMDAR Encephalitis a Rare Genetic Disease?

Similar to other autoimmune diseases (e.g., multiple sclerosis—MS), anti-NMDAR encephalitis has an increased incidence in young women compared with men. Moreover, these disorders share the same progression course, that can be the progressive or relapse-remitting type. Similar with MS, anti-NMDAR encephalitis is not reported to have a clear hereditary background. Nowadays, a clear mechanism is not fully understood, but genetics may play a role in the susceptibility of these disorders. We ask if they are similar to other autoimmune diseases—there is an increased genetic risk of developing anti-NMDAR encephalitis in response to environmental or bacterial/viral triggers.

### 4.1. HLA-Related Genetic Influences in Anti-NMDAR Encephalitis

For the human leukocyte antigen (HLA)-associated risk of anti-NMDAR encephalitis, human leucocyte antigen (HLA) alleles encode antigen presenting proteins that are involved in the susceptibility to or protection from autoimmune diseases [26,27]. These proteins present antigens to T cells and enhance immune responses. HLA molecules as part of the major histocompatibility complex (MHC) are heterodimers formed by two types of chain (the Alpha and Beta chain) [28]. These chains are encoded by different HLA loci (e.g., DR or DQ) with a high degree of polymorphism. Many autoimmune diseases display a strong HLA association. To date, for NMDAR encephalitis, research studies focused on the discovery of a link between different HLA types and individual predisposition to diseases. Recent studies report that there is a specific HLA haplotype that can influence the risk for anti-NMDAR encephalitis by blood–brain-barrier (BBB) disruption and the triggering of an autoimmune response [29,30]. The classical HLA-DRB1 allele (DRB1 16:02 and 15:02) and HLA-DQB1 (e.g., HLA-DQB1 11:02 or HLA-DQB1 05:02) [31] are associated with autoimmune diseases including anti-NMDAR encephalitis [32,33,34]. Some of HLA types display increased racial and ethnic disparities, such as HLA-DRB1 allele-16:02, which was reported to have a strong association with an increased risk for anti-NMDAR encephalitis and has an impact on the therapeutic response in Asian populations (e.g., Chinese Han) [35]. In contrast, in a European cohort, no consistent association of anti-NMDAR encephalitis with a particular HLA allele was reported, only a weak association with the HLA class II region, while the HLA-DQB1 allele displays an inconsistent association across studies with a moderate evidence strength. One possible explanation of this discrepancy can be due to the small and homogenous study cohort.

A genome-wide association study (GWAS) on a multiethnic cohort aimed to identify genetic risk factors for anti-NMDA receptor (anti-NMDAR) encephalitis that belongs to the HLA system [29]. This study reported that human leukocyte antigen (HLA) and killer-cell immunoglobulin-like receptor (KIR) gene polymorphism may influence the anti-tumor and immune response to infections [29]. New HLA alleles are found to be potentially pathogenic in anti-NMDA encephalitis, such as HLADRB1 01:01, HLADRB1 11:01, and HLA DRB1 16:02 [29,31]. In addition, the copy number variation (CNV) of a gene involved in the KIR-mediated immune response (CNV of KIR2DL5B) and two gene alleles (KIR2DL5B and KIR2DL5B) was identified [29].

A gap of knowledge that must be further investigated is that the HLA association seems modest compared with other autoimmune diseases and the immune system response in anti-NMDAR encephalitis might be only in part controlled by *HLA* genes. Moreover, most studies identifying HLA alleles are largely derived from an ethnically homogeneous cohort. To address this gap, multicentric and multiethnic cohort studies are mandatory for relevant HLA allele patterns that allow us to have a more globally representative profile of the HLA association with anti-NMDAR encephalitis susceptibility, progression, and therapeutic management.

### 4.2. Non-HLA Genetic Pattern in Anti-NMDAR Encephalitis

In light of this, the *HLA* genetic factors are the autoimmunity core, but the non-HLA genetic factor might influence the genetic risk and therapeutic response. To date, the non-HLA gene’s role in anti-NMDAR encephalitis is less characterized. For the first time, recent studies on autoimmune encephalitis genetics report that the non-HLA gene is involved in the diseases’ molecular mechanism. Gene mutation, such as single-nucleotide polymorphism (SNP), can increase the risk of developing anti-NMDAR encephalitis. A genome-wide association study (GWAS), aimed at identifying genetic risk factors for anti-NMDA receptor (anti-NMDAR) encephalitis, performed colocalization and gene variant analysis to identify causal genes or gene variants [30]. These variants and genes did not belong to the HLA system. In this genomics study, performed by Tietz and colleagues, two significant genetic loci located on chromosomes 15 and 11 were identified as potential risk factors for anti-NMDAR encephalitis (Table 1). Other genes related to immune system checkpoints or the cytokine signaling pathway which involve BBB could be key factors in anti-NMDAR encephalitis.

The lysosomal acid phosphatase 2 (ACP2) gene encodes an enzyme from the lysosomal membrane which hydrolyzes orthophosphoric monoesters to alcohol and phosphate. About 37 single-nucleotide variants (SNVs) of this gene are reported according to clinvar, but no clear pathological association with immune response was found [30]. ACP2 is involved in the interaction between the host and pathogens, such as viruses [36]. The ACP2 gene involved in immune regulation and the inflammatory signaling pathway may influence antigen processing and potentiate autoantigen production. The mutation of the *ACP2* gene may be used in personalized therapies and can sustain the potential benefit of anti-inflammatory therapy.

Nuclear Receptor Subfamily 1 Group H Member 3 (*NR1H3*) genes were reported to be linked with the risk of anti-NMDA receptor (anti-NMDAR) encephalitis. The NR1H3 gene is involved in blood–brain-barrier integrity as a modulator of macrophage function [37,38]. The *NR1H3* gene is linked with an increased risk of MS [39,40]. Together with the *ACP2* gene, it can guide the anti-inflammatory therapy in anti-NMDAR encephalitis.

Other genes such as the mitogen-activated protein kinase (*MADD*) gene is involved in many neurological disorders [41,42] and can be potentially involved in anti-NMDAR encephalitis development [30]. The MADD is a signaling molecule with a role in the tumor-necrosis factor alpha (TNFalpha) pathway and vesicular trafficking with degranulation defects in MDD-deficient cells [43,44]. Moreover, Damage Specific DNA Binding Protein 2 (DDB2) is involved in DNA repair machinery, cancer development, and the cancer response to chemotherapy [45]. DDB2 acts by targeting other proteins for degradation. A high level of the *DDB2* gene activates the immune response by T-cells and macrophage activation [30,36]. Nowadays, more data about DDB2 are still needed in order to understand how they act and how they can be targeted for the treatment of autoimmune diseases, such as anti-NMDAR encephalitis. Moreover, *C11ORF49/CSTPP1*, a gene that encodes a protein with multiple functions that act as regulator of the tubulin polyglutamylase complex and stabilize it, can be useful as a potential risk factor for anti-NMDAR encephalitis.

Interestingly, Tietz and colleagues report the *LRRK1* gene as “the best candidate” among non-HLA genes for anti-NMDAR encephalitis and provide strong evidence for this [30]. These results come from an unbiased, high-throughput GWAS analysis of genetic variants across the genome of anti-NMDAR patients compared with healthy controls. This study was performed on 212 samples from patients with anti-NMDAR encephalitis. The association *p* value was 1.18 × 10^−8^ and exceeds the conventional threshold for GWAS significance, *p* < 5 × 10^−8^ [45].

The *leucine-rich repeat kinase (LRRK) 1* gene is a large multidomain protein involved in B-cell function and NF-kB pathway activation, resulting in a pro-inflammatory response [46]. The LRRK1 mutation was reported to be involved in optic nerve atrophy [47], but its role in anti-NMDAR encephalitis development remains unclear. The *LRRK1* gene expression is increased in Epstein–Barr virus (EBV)-transformed lymphocytes and whole blood according to the Genotype-Tissue Expression portal (GTEx). A recent study on the LRRK1 structure emphasizes the important differences between the LRRK1 and LRRK2 gene, and further research is mandatory in order to better understand its mechanism of activation [48].

A gene locus provides information for the correct gene expression (e.g., set of products) in a time and spatial way. The identified chromosome 15 loci (near the *LRRK1* gene) are responsible for B-cell development, activation, and survival. The dysfunction of this gene loci could lead to gene expression changes in anti-NMDAR encephalitis, such as the increased survival of autoreactive B-cells and increased anti-NMDAR antibody production (GluN1 subunit) and pro-inflammatory cytokine production, while the chromosome 11 loci findings have an impact on the inflammatory gene expression balance, immune regulation, and antigen processing. The dysfunction of the gene expression located on chromosome 11 might contribute to a pro-inflammatory environment and inefficient immune regulation. This environment triggers anti-NMDAR antibody production and leads to brain function disruption, which leads to neurological and psychiatric symptoms (e.g., psychosis, seizures, motor impairment such as catatonia, etc.) as the hallmark of the diseases.

**Figure 1:**
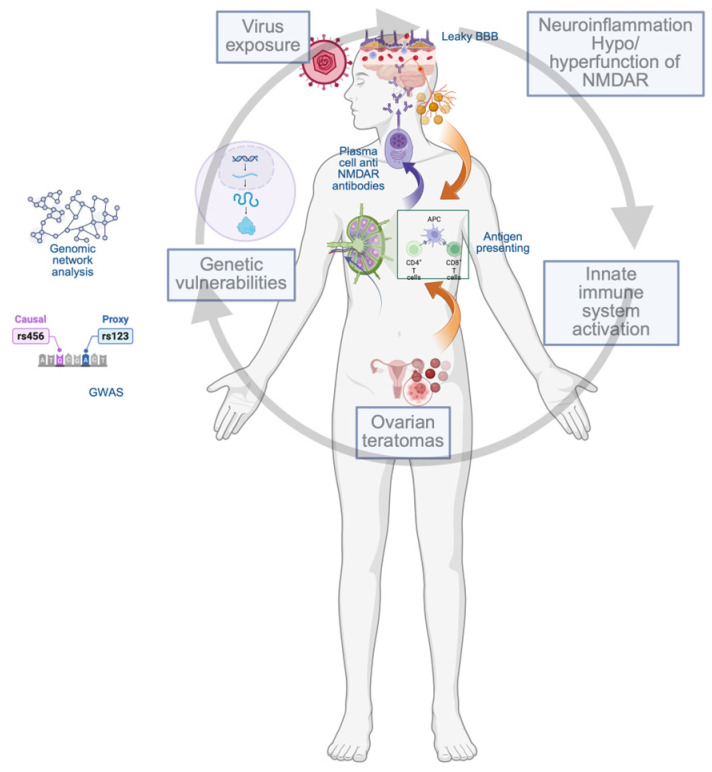
Anti-NMDAR genomic vulnerability as missing piece of molecular mechanism. Leaky *BBB* is a hallmark of CNS homeostasis disruption and can appear due, first, to direct *viral invasion* of endothelial cells, and viral-induced endothelial/glial cell death. Second way to open the CNS “gate” is mediated by innate/adaptative immune system. Infected neuronal cells present self-antigens to antigen presenter cells (APCs) and/or viral cells can mimic self-antigen. These events trigger antibody production, inflammatory cytokine production, and matrix metalloproteases synthesis (MMPs) that favor leukocyte and antibody extravasation. Tumor cells act by activation or not of immune cells. Immune cells are activated by onconeural-type antigen or can act without immune cell activation by express immune checkpoint ligands (e.g., PDL1 and/or CTLA4). These events are potentiated by genomic changes that increase brain vulnerability to neuroinflammation and oxidative stress, and maintain antiNMDAR’s antibody production in a vicious cycle. Created in BioRender. b, a. (accessed on 8 April 2025) https://BioRender.com/fzqq8bt.

These are key findings for the development of better anti-NMDAR encephalitis therapeutic strategies, and they help us move forward from standard therapy to personalized therapy by using genetic stratification in therapeutic decisions and promoting routine genetic screening. The identification of the risk variant gene located on chromosome 15 can sustain an early B-cell-targeted therapy as an on-time intervention, and an increased frequency of relapse monitoring (due to the prediction of increased autoantibody production) for the better control of the diseases’ progression. In addition, patients with an identified risk due to the dysregulation of the gene expression linked with chromosome 11 loci can have an increased benefit from early immunotherapy. However, epigenetic markers such as the miRNA pattern integrated with chromosomal loci mapping could be the missing piece from the complex puzzle of anti-NMDAR encephalitis risk and management. However, genomic vulnerabilities may precipitate disease events and impact anti-NMDAR immunopathogenesis by promoting autoantibody production (Figure 1). However, the involvement of this gene and the impact on disease management remain to be further clarified.

Apart from the risk associated with loci outside the HLA region, some studies report other genes associated with anti-NMDAR encephalitis. These genes belong to different signaling pathways. One of these pathways is related with *B-cell development*, *activation, and survival*. *B-cell* genes are upregulated in anti-NMDAR encephalitis (the *CD19, FCRL1*, and *CXCR5* gene) and create a distinct gene pattern [31].

Some rare gene variants related to immune regulation pathways, such as the complement pathway, might play an important role in anti-NMDA encephalitis risk (e.g., non-functional inherited *C4B* gene mutation) [49]. But these polygenic studies are limited and seems to need large multiethnic studies to confirm. Interestingly, epigenetic factors such as microRNA (e.g., let-7 family miRNA dysregulation), a small non-coding RNA that controls gene expression, could have an impact on the diseases’ onset and therapeutic response without changes in the DNA sequences. A study by Wang identifies a genetic predisposition linked to epigenetic regulation (miRNA expression pattern) and tumors’ association (especially ovarian teratomas) with the diseases [50]. To date, no functional validation of IRF7 and GRIN 1 genotypic–phenotypic association was reported. In the future, this validation needs to be proven.

## 5. Clinical Features of Anti-NMDA Receptor Encephalitis

One of the striking aspects of anti-NMDA receptor encephalitis is the broad range of symptoms, which often evolve in a characteristic multiphasic pattern. Early symptoms are frequently psychiatric in nature, including acute behavioral changes, agitation, hallucinations, and delusions. These neuropsychiatric manifestations often lead patients to seek psychiatric evaluation before the subsequent development of more overt neurological deficits [51]. According to a systematic analysis, 633 patients with anti-NMDAR encephalitis experienced behavioral (80.3%), neurological (75.5%), seizure (63.2%), autonomic (51.3%), psychotic (45.8%), and cognitive (43.9%) symptoms over the course of their disease [7]. Aberrant behaviors (such as agitated, weird, or sobbing) accounted for 80.3% of the most prevalent psychiatric symptoms, followed by aberrant speech (such as silent or echolalia) at 50.4%, catatonia (32.7%), hallucinations (31.3%), sleeplessness (23.4%), mood (24.5%), and delusions (20.5%) [7]. We found that 23 (4%) of the 571 patients with anti-NMDAR encephalitis in the prior review experienced isolated mental symptoms [19].

As the disease progresses, patients may develop seizures, dyskinesias (such as orofacial or limb dyskinesias), movement disorders, and autonomic instability (e.g., tachycardia, blood pressure lability, and hyperthermia) [52]. Further progression can result in a decreased level of consciousness and even coma if not recognized and treated promptly. In children, the presenting symptoms may lean more heavily toward abnormal movements, seizures, and behavioral disturbances, sometimes making the diagnosis challenging. As initially documented by Iizuka et al. [53], patients go through multiple phases of disease and recovery. They said that prodromal, psychotic, unresponsive, hyperkinetic, and gradual recovery were the five stages that typical patients’ clinical courses passed through, in that order. Within two weeks prior to being admitted to the hospital, most patients in the prodromal phase had a headache, a low-grade fever, or an illness that resembled a virus [9,54].

Patients experienced cognitive decline, emotional disturbances (such as apathy, lack of emotion, depression, loneliness, or fear), and prominent symptoms of schizophrenia, such as compulsive ideation, disorganized thinking, delusions, hallucinations, and loss of self-awareness, during the psychotic phase [55,56]. Most patients experienced seizures, decreased verbal output, and decreased consciousness throughout this phase [9,57]. During the unresponsive phase, patients exhibited catatonia-like muteness, akineticity, and an inability to respond to spoken orders while maintaining ocular openness [58]. Additionally, they displayed catalepsy-like symptoms, athetoid dystonic postures, echo phenomena, and strange and inappropriate smiling [9,58]. Patients gradually acquired athetoid dystonic finger postures and orolingual dyskinesia, including lip licking and biting, during the hyperkinetic phase [58]. Most patients at this stage experienced autonomic instability symptoms, such as diaphoresis, bradycardia or tachycardia, labile blood pressure, and hyperthermia. A few patients also experienced central hypoventilation, necessitating ventilator assistance [9,58,59]. Following immunotherapy or anti-inflammatory medication, this illness usually has a slow and protracted healing period [2]. The reduction in anti-NMDAR antibody titers may be linked to the alleviation of neurological symptoms, and over 75% of patients ultimately have a significant recovery that happens in the opposite sequence of symptom progression [2,60]. Nevertheless, a recent study found no correlation between psychiatric symptoms and antibody titers [61].

## 6. Anti-NMDA Encephalitis Diagnostic Pearls

Clinical suspicion is paramount in patients—especially adolescents and young adults—with a combination of new-onset psychiatric symptoms, seizures, movement disorders, and/or autonomic dysregulation. Investigations that aid diagnosis include serology and cerebrospinal fluid (CSF) analysis: The detection of anti-NMDAR antibodies in the CSF is the gold standard [1]. Serum testing can also be performed, but CSF testing is often more sensitive and specific. Lymphocytic pleocytosis, elevated protein, and oligoclonal bands can be found in the CSF. A positive serum or CSF sample test for antibodies against the NMDA receptor subunit is necessary in order to confirm the clinical diagnosis of anti-NMDA receptor encephalitis. The question of whether serum or CSF is better for testing is still up for debate. While Irani and Vincent [59] state that the serum levels of anti-NMDA receptor antibodies were comparable to or higher than those of CSF, Dalmau advises testing for both [2]. There is a strong correlation between the antibody titer and the disorder’s clinical symptoms [9]. Even if the test for anti-NMDA receptor encephalitis is now a little slow, it is reasonably priced; thus, it should be taken into consideration for any patient who presents with sudden onset mental symptoms together with strange movements or atypical features. About 80% of cases have been reported to have CSF abnormalities, which include CSF-specific oligoclonal bands, a normally or slightly elevated protein content, and mild lymphocytic pleocytosis [9,62].

Neuroimaging: Magnetic resonance imaging (MRI) of the brain may reveal T2/FLAIR hyperintensities in the medial temporal lobes or other cortical or subcortical regions, though a significant subset of patients may have normal MRI findings, particularly early in the disease [63]. According to reports, 70% of brain magnetic resonance imaging tests are normal [15]. The rest may have hyperintensities in several places, including the brainstem, frontobasal, insular, basal ganglia, cerebellum and cerebral cortex, and hippocampus [1].

Electroencephalography (EEG): EEG is almost universally abnormal, typically showing diffuse slowing or focal epileptiform discharges. A classic (though not pathognomonic) finding is the so-called “extreme delta brush”, which can support the clinical suspicion in the right context [64]. Electroencephalograms (EEGs) typically exhibit sluggish continuous rhythmic activity or non-specific slowing during the catatonic phase of disease [65]. Since 90% of individuals with anti-NMDA receptor encephalitis exhibit non-specific slowing at some point throughout their illness, an EEG is particularly useful when attempting to differentiate between encephalitis and a main psychiatric problem [15].

Tumor screening: Given the association with teratomas and other malignancies, all patients should undergo age- and sex-appropriate screening, including a pelvic ultrasound or MRI, and, in some cases, whole-body imaging (e.g., PET/CT). While other studies have been reviewed, they are not currently likely to support clinical practice. Results from positron emission tomography have been inconsistent, with some suggesting cerebral hypometabolism [66]. This suggests subcortical hypermetabolism, which contradicts the findings from other researchers [67].

Anti-NMDA receptor encephalitis can be easily misdiagnosed. Depending on whether mental symptoms come before neurological features, as is frequently the case, the disorder may manifest in the realm of either a psychiatrist or a neurologist.

### 6.1. The Neurological Red Flags

Viral encephalitis, cerebral vasculitis, other types of autoimmune encephalitis, and encephalitis lethargica are frequently included in neurological differential diagnoses [68]. Dyskinetic motions might be confused with tardive dyskinesia or seizure activity. Additionally, patients may exhibit strange stereotypes. Orofacial dyskinesia and repetitive stereotypies can be confused with seizures [69]. Another possible misdiagnosis for the seizure-like dyskinetic movements is status epilepticus, which is known to occur in 6% of cases [69]. Two cases where status epilepticus was suspected but a video EEG showed encephalopathy were reported by Dericioglu et al. [67]. This prevented aggressive treatment with intravenous anesthetics. Therefore, when status epilepticus is suspected, care should be taken when interpreting these movements unless they are explained by a video EEG [70].

### 6.2. Mental Health Red Flags

In the early stages of illness, psychiatric differential diagnoses are typically the main differential. Due to the presence of delusions, hallucinations, and catatonic symptoms, new-onset psychosis is usually included in the literature as the most prevalent first diagnosis. According to recent research, up to 5–10% of first-onset psychosis may be caused by this disorder or other related autoimmune diseases, which can present with a more classic schizophrenia appearance [11,18]. In a prospective trial, Zandi et al. [18] searched for NMDA receptor antibodies in a group of 46 individuals with first-episode psychosis; only two of them tested positive. According to the scientists, there were no clinical characteristics that set these people apart from the other psychotic members of the cohort. There have also been reports of “postnatal psychosis” cases linked to ovarian pathology that remarkably resemble anti-NMDA receptor encephalitis [71]. When antipsychotic drugs are taken, the presence of rigidity and altered consciousness, which are typical in anti-NMDA receptor encephalitis, may also raise the possibility of a neuroleptic malignant syndrome diagnosis. Given that these illnesses are obviously not mutually exclusive, this could pose a diagnostic conundrum as well as a therapeutic difficulty in clinical practice.

## 7. Therapeutic Strategies in Anti-NMDA Receptor Encephalitis

Early and aggressive immunotherapy is the cornerstone of management for anti-NMDA receptor encephalitis and has been correlated with improved outcomes. The therapeutic approach often proceeds in stages:

First-line therapy usually involves high-dose corticosteroids (e.g., intravenous methylprednisolone), intravenous immunoglobulins (IVIG), and/or plasmapheresis (plasma exchange). These therapies aim to reduce antibody production and remove circulating antibodies from the bloodstream [72]. The treatment beginning may be significantly hampered by behavioral disturbances, which frequently necessitate sedated patients to administer plasma exchange.

Second-line therapy may be considered in patients who do not respond adequately to first-line therapies. Options include rituximab and cyclophosphamide, both of which target B cells to limit autoantibody production. These are frequently necessary for people without tumors or those who receive a delayed diagnosis [73]. Alemtuzumab was used in an 8-year-old child with a favorable result, according to Liba et al. [70].

Tumor resection is essential if an underlying neoplasm (e.g., ovarian teratoma) is identified. The prompt removal of the tumor may hasten clinical improvement and reduce the risk of relapse.

Psychiatric symptoms often require adjunctive treatment with atypical antipsychotics or benzodiazepines to manage agitation, psychosis, and sleep disturbances. Supportive care measures—such as seizure management, nutritional support, and monitoring for autonomic dysfunction—are also critical in the acute phase.

Antipsychotics have been used to treat behavioral and psychotic symptoms, both normal and abnormal, in the short term. It should be mentioned that using antipsychotics can make matters more complicated, especially before a definitive antibody diagnosis is made. Autonomic stiffness and instability can be confused with neuroleptic malignant syndrome. Furthermore, corticosteroid use can be mistaken for steroid-induced psychosis. Benzodiazepines, trazadone, and clonidine have all been effectively used to reverse sleep disturbances [74]. Benzodiazepines are commonly used to treat catatonic symptoms. Although not much is known about lorazepam’s effectiveness in treating anti-NMDA receptor encephalitis, doses of up to 20–30 mg per day have been utilized to treat catatonia symptoms [75]. Anti-NMDA receptor encephalitis has not received much research on electroconvulsive therapy (ECT), despite being the gold standard for treating catatonia when benzodiazepines are not effective. There have been case reports of anti-NMDA receptor encephalitis exhibiting a catatonic clinical response [76]. Remarkably, it has been shown that, in animal models of ECT activity, the NMDA receptor is upregulated due to an increase in the messenger ribonucleic acid (mRNA) of the NMDA subunits NR2A and NR2B [77].

## 8. Long-Term Outcome in Anti-NMDA Receptor Encephalitis

With early recognition and aggressive treatment, most patients with anti-NMDA receptor encephalitis can achieve good functional recovery. However, recovery is often protracted, with improvement continuing over months to years [1,16]. Relapses can occur in up to 12–25% of cases, often associated with a reduction or cessation in immunosuppressive therapy or incomplete tumor resection [19].

Long-term neurological sequelae can include cognitive deficits, persistent psychiatric symptoms, or movement disorders, underscoring the importance of a close follow-up with a multidisciplinary team (neurology, psychiatry, and sometimes gynecology or oncology for tumor surveillance).

Several prognostic factors are involved. Finke et al. [75] found that adult patients with anti-NMDA receptor encephalitis who received immunomodulatory therapy within three months of the onset of the disease had a better cognitive outcome in terms of the length of illness and treatment outcome when compared to those who received treatment later or not at all. The ideal period of time between the beginning of symptoms and treatment has not yet been established, despite the authors’ suggestion that postponing treatment could result in irreversible hippocampus damage [78]. Additional outcome predictors that have been found include less severe symptoms, no need for intensive care unit hospitalization, early immunotherapy commencement, and, if present, tumor excision [15,79].

## 9. Limitation and Future Perspectives

Starting with the Human Genome Project that began in 1990, which provided the first information about the sequence of the human genome, we constantly gain information about the genetic profile of the human body in health and disease. More than 80% of rare disorders are reported to have a genetic origin and usually are primarily linked with a single gene variant [80]. The implementation of high-resolution genetic technologies allows us to switch the research studies’ strategy from the genome to genomics. Nowadays, genomics studies of disorders associated with intellectual disabilities identified different genetic variations, such as copy number variations (CNVs), tandem repeats, indels, or short variations, that play an important role in the development of diseases including rare disorders [81]. Moreover, we hypothesize that anti-NMDA receptor encephalitis pathophysiology may involve gene variants that predispose us to CNS inflammation and this risk can be polygenic. All these changes can be identified by the whole-genome sequencing (WGS) technique that assesses both the coding and non-coding region of genome. Using this approach, we identify only one study by Malik and colleagues that significantly identified the involvement of polygenic risk [82]. To date, we have identified several limitations in the topic of research studies, such as the limited cohort included in the study or the lack of validation by other methods. However, there exists no universal HLA marker globally and several risk loci outside of the HLA region were reported, but these results are only preliminary.

## 10. Conclusions

Anti-NMDA receptor encephalitis represents a unique and multifactorial autoimmune encephalitis that straddles the intersection between neurology, psychiatry, and oncology. Its heterogeneous clinical presentation underscores the necessity of maintaining a high index of suspicion. Progress in diagnostic testing including genomics and the establishment of standardized treatment protocols will enhance diagnostic accuracy and patient outcomes. While anti-NMDAR encephalitis is not classified as a genetic disorder, innate immune-related genetic factors and specific gene variants may influence diseases’ susceptibility and response to therapy. Future research in immunogenetics has the potential to refine treatments, accelerate recovery, and reduce relapse rates.

## Figures and Tables

**Table 1:** Non-HLA genes related to autoimmune encephalitis.

Gene Function	Genes	Mutation Type	Gene Localization
Pro-inflammatory states and impaired immune system regulation			
ATP/ITP signalling pathway and antigen processing	*ACP 2*	Germline mutation Potential causative mutation	Chromosome 11 [1]
Regulation of inflammation and lipid metabolism	*NR1H3*	Potential causative mutation	Chromosome 11 [1]
Connect TNFR1 with MAP kinase activation and arachidonic acid release [2]/TNFalpha signalling pathway	*MADD*	Germline/Potential causative	Chromosome 11 [1]
Innate immune response against viruses; anti-tumor factor	*IRF7*	Potential causative polymorphism	Chromosome 11 [6]
Innate immune response through interferon (IFN) signaling pathway	*IFIH1*	Potential causative missense mutation	Chromosome 2 [11]
Th1 cell function, activate IFN-gamma and CXCR3; regulator of antiviral B-cell responses [8]	*TBX21*	Potential causative polymorphism	Chromosome 17 [6,7]
B-cell function regulation	*BANK1*	Potential causative polymorphism	Chromosome 4 [6]
Immune cell development, activation, and survival			
B-cell development and survival intracellular trafficking [5]	*LRRK1*	Potential causative	Chromosome 15 [1]
Cytoskeletal organization, nuclear morphology, and cilium disassembly [4]	*C11ORF49/CSTPP1*	Potential causative	Chromosome 11 [1]
Chromosome 9 [9]	*GRIN1*	Pathogenic mutation	Intellectual disability; NMDA receptor trafficking and function [10]

## Data Availability

Not applicable.
